# Erste zugelassene rheumatologische DiGA für die axiale Spondyloarthritis

**DOI:** 10.1007/s00393-026-01826-0

**Published:** 2026-05-07

**Authors:** Martin Krusche

**Affiliations:** Sektion Rheumatologie, Zentrum für Innere Medizin, III. Medizinische Klinik und Poliklinik, Universitätsklinikum Eppendorf, Martinistraße 52, 20246 Hamburg, Deutschland

## Abstract

Bewegungstherapie ist ein zentraler Bestandteil der Behandlung der axialen Spondyloarthritis (axSpA) und wird in der deutschen S3-Leitlinie unabhängig von der medikamentösen Therapie als Basismaßnahme empfohlen. In der Versorgungspraxis wird eine kontinuierliche physiotherapeutische Betreuung jedoch häufig nicht ausreichend umgesetzt. Digitale Gesundheitsanwendungen (DiGA) könnten dazu beitragen, leitliniengerechte Bewegungstherapie im Alltag besser zu unterstützen.

Mit der App Axia – für axiale Spondyloarthritis steht erstmals eine dauerhaft im DiGA-Verzeichnis des Bundesinstituts für Arzneimittel und Medizinprodukte (BfArM) gelistete digitale Therapie für eine entzündlich-rheumatische Erkrankung zur Verfügung. Die Anwendung kombiniert evidenzbasierte Bewegungstherapie mit edukativen Inhalten und strukturierten Selbstmanagement-Elementen und richtet sich an Patientinnen und Patienten mit axSpA.

Die klinische Wirksamkeit wurde im prospektiven, randomisiert-kontrollierten Bechterew-App-Trial untersucht. Insgesamt wurden 200 Patientinnen und Patienten mit gesicherter axSpA unter stabiler medikamentöser Therapie randomisiert; 186 schlossen die 12-wöchige Studie vollständig ab. Die Interventionsgruppe nutzte Axia zusätzlich zur Standardversorgung, während die Kontrollgruppe ausschließlich Standardversorgung erhielt.

Nach 12 Wochen zeigte sich ein signifikanter Vorteil zugunsten der Interventionsgruppe in allen koprimären Endpunkten: Krankheitsaktivität (BASDAI −1,51), Funktionsfähigkeit (BASFI −1,14) und krankheitsspezifische Lebensqualität (ASQoL −2,30; jeweils *p* < 0,001). Auch in den Responderanalysen zeigte sich ein höheres Ansprechen (ASAS20: 51 % vs. 9 %; ASAS40: 23 % vs. 3 %).

Sicherheitsrelevante Ereignisse wurden nicht berichtet.

Die Ergebnisse zeigen, dass eine digital vermittelte Bewegungstherapie bei axSpA klinisch relevante Verbesserungen erzielen kann. Damit steht erstmals eine evidenzbasierte, verordnungsfähige digitale Ergänzung zur leitliniengerechten Therapie der axSpA zur Verfügung.

Krankengymnastik und strukturierte Physiotherapie sind zentrale Bausteine der Therapie der axialen Spondyloarthritis (axSpA). Die deutsche S3-Leitlinie empfiehlt regelmäßige, aktive Bewegungstherapie als Basismaßnahme (unabhängig von der medikamentösen Behandlung), da sie Krankheitsaktivität, Funktion und Beweglichkeit verbessert [1]. In der Versorgungspraxis wird physikalische Therapie jedoch nicht flächendeckend und kontinuierlich umgesetzt [2], unter anderem aufgrund struktureller Engpässe, organisatorischen Aufwands und einer stärkeren Fokussierung auf medikamentöse Therapien.

Ein vielversprechender Ansatz, um diese Versorgungslücke zu schließen, sind digitale Behandlungsmöglichkeiten [3]. Mit der dauerhaften Aufnahme von *Axia – für axiale Spondyloarthritis* in das Verzeichnis digitaler Gesundheitsanwendungen (DiGA) des Bundesinstituts für Arzneimittel und Medizinprodukte (BfArM) steht [4] hier erstmals eine digital basierte, randomisiert geprüfte Intervention für eine entzündlich-rheumatische Erkrankung in der Regelversorgung zur Verfügung.

Die App richtet sich an Patient:innen mit axSpA und kombiniert leitlinienorientierte Bewegungstherapie und Physiotherapie mit edukativen Inhalten und strukturierten Selbstmanagement-Elementen [5]. Konkret umfasst *Axia* mehrere Module, die zur Durchführung physiotherapeutischer Maßnahmen (insbesondere Widerstandsübungen, Dehnungen, Stabilisationsübungen und Atemübungen) anleiten. Die Anleitung erfolgt videobasiert sowohl vor Beginn der Übung als auch während ihrer Durchführung durch gesprochene Hinweise (Abb. 1):

- Tägliche (ähnlich wie bei klassischer Physiotherapie in Einzelbehandlung) auf den Nutzer angepasste Übungsprogramme
- „Sofort-Hilfe“-Übungsmodul (physiotherapeutische Mobilisationsübungen der Wirbelsäule gegen akute Schmerzen oder Morgensteifigkeit)
- Tägliche Routinen (i. d. R. Integration von 1–3 passenden physiotherapeutischen Übungen in alltägliche Gewohnheiten oder Verknüpfung an andere feste Ankerpunkte im Alltag)

Weitere Module dienen der Steigerung der Alltagsaktivität und körperlichen Betätigung:

- Integrierter Schrittzähler (mit Option zur Smartwatch-Verknüpfung) mit täglichem Schrittziel
- Tägliche Routinen (einzelne Routinen dienen der generellen Aktivitätssteigerung, z. B. täglich einen kurzen Spaziergang machen)
- Erfassen von Sporteinheiten (Dauer, Intensität, Sportart)
- Kardiorespiratorische Trainingsmöglichkeiten

Die Applikation wurde in Kooperation zwischen dem Universitätsklinikum Würzburg, dem Start-up Applimeda GmbH und der Deutschen Vereinigung Morbus Bechterew entwickelt. Die physiotherapeutischen Inhalte wurden mit Physiotherapeuten der Gesellschaft medizinischer Assistenzberufe für Rheumatologie e. V. (GMAR) entwickelt.

**Abb. 1:**
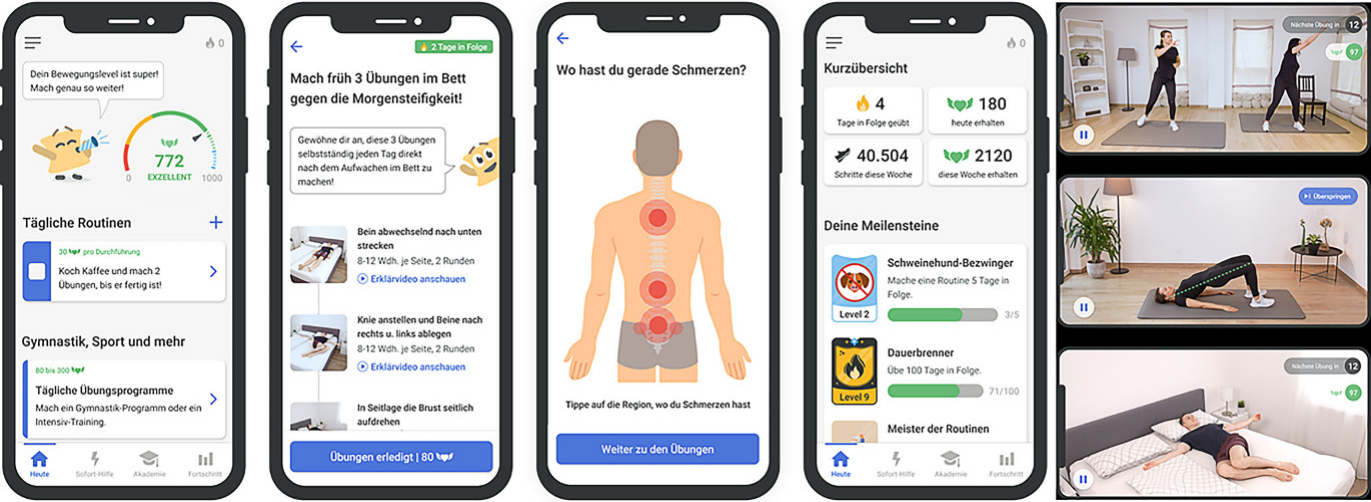
Beispielbild der DiGA *Axia*. (Mit freundl. Genehmigung von © Applimeda GmbH)

## Evidenzlage

Die klinische Wirksamkeit der digitalen Anwendung wurde in einer prospektiven, randomisiert-kontrollierten Studie zur Effektivität einer App-basierten Intervention bei Patienten mit axialer Spondyloarthritis (kurz: „Bechterew-App Trial“) untersucht. Die Ergebnisse der Arbeit wurden jüngst in den *Annals of Rheumatic Diseases* publiziert [5].

Insgesamt wurden in der Studie 200 Erkrankte mit gesicherter axSpA unter stabiler medikamentöser Therapie randomisiert. Die Interventionsgruppe nutzte Axia zusätzlich zur Standardversorgung über 12 Wochen, während die Kontrollgruppe ausschließlich Standardversorgung erhielt. 186 Personen schlossen die Studie vollständig ab.

Als koprimäre Endpunkte fungierten die Veränderungen der Krankheitsaktivität (gemessen am Bath Ankylosing Spondylitis Disease Activity Index [BASDAI]), der Funktionsfähigkeit (Bath Ankylosing Spondylitis Functional Index [BASFI]) sowie der krankheitsspezifischen Lebensqualität (Ankylosing Spondylitis Quality of Life Questionnaire [ASQoL]).

Nach 12 Wochen zeigten sich in allen drei koprimären Endpunkten signifikante Vorteile zugunsten der Interventionsgruppe. Für den BASDAI ergab sich eine adjustierte mittlere Differenz von −1,51 Punkten (*p* < 0,001). Auch für den BASFI (−1,14 Punkte; *p* < 0,001) und im ASQoL (−2,30 Punkte; *p* < 0,001) ließen sich deutliche, Baseline-adjustierte Verbesserungen durch die App-Nutzung nachweisen. Die Analyse der Therapieansprechraten zeigten weiterhin, dass signifikant mehr Teilnehmende in der Interventionsgruppe Verbesserungen erzielten, welche die vordefinierten minimal klinisch wichtigen Unterschiede (MCID) überschritten, verglichen mit der Kontrollgruppe (BASDAI: 67 % vs. 18 %; BASFI: 67 % vs. 22 %; ASQoL: 60 % vs. 28 %; alle *p* < 0,001).

Das ASAS20-Ansprechen wurde von 51 % der Patientinnen und Patienten in der Interventionsgruppe erreicht, verglichen mit 9 % in der Kontrollgruppe (*p* < 0,001). Das ASAS40-Ansprechen betrug 23 % vs. 3 % (*p* < 0,001). Insgesamt wurden in der Studie 4 schwere unerwünschte Ereignisse (SAEs) berichtet. In der Interventionsgruppe gab es eine SAE (epileptische Anfall) und in der Kontrollgruppe 3 (Prostatakarzinom, schwere Infektion und Hüft-TEP). Geräteassoziierte unerwünschte Ereignisse traten nicht auf.

Die Daten zeigen damit konsistente, statistisch signifikante und klinisch relevante Verbesserungen zentraler patientenrelevanter Parameter innerhalb eines 12-wöchigen Beobachtungszeitraums und bildeten die Grundlage für die Zulassung durch das BfArM.

## Verordnung und Kosten

*Axia* ist gemäß § 33a SGB V extrabudgetär und für die vertragsärztliche Versorgung budgetneutral verordnungsfähig. Die Verschreibung erfolgt regulär über ein Muster-16-Rezept unter Angabe der Pharmazentralnummer (PZN 20516705). Nach Ausstellung wird das Rezept bei der zuständigen Krankenkasse eingereicht. Nachdem die Krankenkasse das Rezept geprüft hat, übersendet diese der Patientin oder dem Patienten einen 16-stelligen Aktivierungscode. Mit diesem Code kann die DiGA nach Herunterladen vom Patienten aktiviert und genutzt werden.

Alternativ zum Muster 16 können DiGAs seit Kurzem auch per E‑Rezept verordnet werden: In diesem Fall sollte jedoch optimalerweise ein Papierausdruck mitgegeben werden, um die Möglichkeit der Einreichung bei der Krankenkasse zu gewährleisten.

Für Ärzt:innen und RFA besteht die Möglichkeit eines kostenlosen Testzugangs. Dieser kann auf https://axia-app.de/fachkreise (Formular am Seitenende) oder per Mail an info@axia-app.de beantragt werden.

Zugelassene ICD-10-GM-Diagnosecodes sind M45 (Spondylitis ankylosans), M46.8 (sonstige näher bezeichnete entzündliche Spondylopathien) sowie M46.9 (entzündliche Spondylopathie, nicht näher bezeichnet). Absolute Kontraindikationen für die Verordnung sind bösartige Neubildungen oder Metastasen an der Wirbelsäule, infektiöse Arthritiden/Spondylopathien, Osteomyelitiden, pyogene Bandscheibeninfektion. Die reguläre Verordnungsdauer beträgt 12 Wochen bzw. 90 Tage. Für die im DiGA-Verzeichnis empfohlene kontinuierliche Nutzung sind daher regelmäßige Folgeverschreibungen erforderlich.

**Abb. 2:**
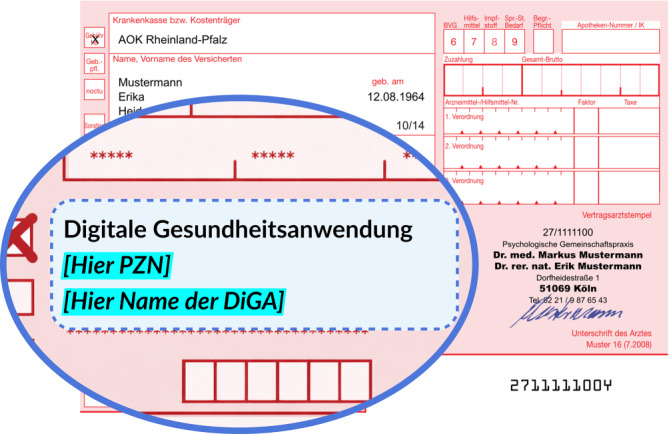
Beispielrezept für eine DiGA

Die Kosten werden von den gesetzlichen Krankenkassen vollständig übernommen; für gesetzlich Versicherte entsteht keine Zuzahlung. Auch die meisten privaten Krankenkassen übernehmen die Kosten vollständig; eine Kostenübernahmeanfrage wird empfohlen. Der im DiGA-Verzeichnis ausgewiesene Bruttopreis liegt im ersten Jahr nach Zulassung bei 599 € und wird ab Februar 2027 durch einen mit dem GKV-Spitzenverband verhandelten, regulären Erstattungsbetrag (erfahrungsgemäß ca. 226 €) abgelöst (Abb. 2).

## Praktische Bedeutung für die rheumatologische Versorgung

Die Studiendaten des „Bechterew-App Trial“ belegen, dass eine strukturierte, digital vermittelte Bewegungstherapie bei axSpA messbare Effekte auf Krankheitsaktivität, Funktion und Lebensqualität erzielen kann.

Damit wird erstmals für eine DiGA im Bereich entzündlich-rheumatischer Erkrankungen ein klarer klinischer Nutzen anhand etablierter Outcomeparameter gezeigt.

Für die Praxis bedeutet dies, dass neben pharmakologischen Strategien eine evidenzbasierte, verordnungsfähige digitale Bewegungstherapie zur Verfügung steht, die leitliniengerechte Bewegungstherapie systematisch unterstützt. Bewegung ist ein zentraler Bestandteil der axSpA-Therapie, ihre kontinuierliche Umsetzung im Alltag ist jedoch häufig limitiert. Die DiGA *Axia* kann hier eine strukturierte Anleitung, regelmäßige Impulse und ein nachvollziehbares Trainingskonzept bereitstellen.

Einschränkend ist jedoch anzumerken, dass bei der Verordnung auch weitere patientenbezogene Faktoren – etwa die digitale Gesundheitskompetenz und eine ausreichende anzunehmende Adhärenz – sowie bereits etablierte Alternativen und individuelle Präferenzen (z. B. Sportverein, Fitnessstudio oder Physiotherapie) kritisch berücksichtigt werden sollten. Über eine Verordnung sollte daher stets im Einzelfall entschieden werden. Darüber hinaus sollte die DiGA-Verordnung analog zur Arzneimittelverordnung dauerhaft ärztlich begleitet und betreut werden. Mit der dauerhaften Aufnahme in das DiGA-Verzeichnis steht somit erstmals eine digital basierte, randomisiert geprüfte Intervention für die axSpA zur Verfügung, die klinisch relevante Effekte zeigt und regelhaft in die Versorgung integriert werden kann.
